# Ecological Analysis of Parking Prices and Active Commuting in US Cities, 2009

**DOI:** 10.5888/pcd13.160097

**Published:** 2016-09-08

**Authors:** Geoffrey P. Whitfield, Arthur M. Wendel, Amy H. Auchincloss

**Affiliations:** Author Affiliations: Arthur M. Wendel, Centers for Disease Control and Prevention, Healthy Community Design Initiative, Atlanta, Georgia; Amy H. Auchincloss, Drexel University School of Public Health, Department of Epidemiology and Biostatistics, Philadelphia, Pennsylvania.

## Abstract

We conducted an ecological study to determine whether parking prices are associated with active commuting across US cities. We obtained parking prices for 107 US cities from the Drexel University Central Business District Public Parking Survey, obtained city prevalence of walking and bicycling to work from the American Community Survey, and used weighted least squares linear regression to explore associations between parking prices and active commuting. After adjusting for several covariates, walking to work was 3.1% higher for every additional dollar charged for off-street daily parking, but only among more densely populated cities, and no such association was detected for bicycling to work. These preliminary results hint at the potential for parking policies to influence commuting mode choice, a link that city planners and public health officials could consider when evaluating parking policies and active transportation behaviors.

## Objective

Transportation-related walking and bicycling is that which occurs while traveling from place to place and is a potential target for increasing physical activity (1,2). Compared with research on the built environment and its relationship to physical activity and active transportation (3,4), research on disincentives for car use, including increased parking prices, is sparse (5). High parking prices might encourage walking and bicycling to avoid payment, particularly in densely populated areas with many destinations in a small area. The purpose of our ecological study was to determine whether parking prices are associated with walking and bicycling to work in US cities.

## Methods

We obtained parking price information from the Drexel University Central Business District Public Parking Survey (2009), the detailed methods of which have been published (6). Parking professionals in 125 cities were contacted; 102 (82%) responded to a questionnaire via Internet or paper. Five provided insufficient information and were excluded, but an additional 10 were included after obtaining data from city websites or secondary data sources (7,8). In all, 107 cities had sufficient data for inclusion in this study. Three variables were hypothesized to be positively associated with prevalence of active commuting in a city: daily off-street parking price, hourly off-street parking price, and maximum hourly on-street (metered) parking price.

We obtained the city-level prevalence of walking to work or bicycling to work (active commuting) from the 2009 American Community Survey (ACS) (9). The ACS survey asks each employed person in a household to report the primary method of transportation used to commute to work in the past week. ACS data are collected year-round to ameliorate seasonal variation. Selected covariates were obtained at the city level from the 2010 decennial census (population density, median age, percentage male, percentage non-Hispanic white, and average household size) and from the 2009 ACS (median family income and the percentage of families at or below poverty level).

Bivariate associations between price variables and active commuting were assessed with Spearman rank correlations. We used weighted linear least squares regression to model the association between daily off-street parking prices (continuous) and walking to work and bicycling to work (separately), adjusted for covariates. The analytic weight was the reciprocal of the standard error of the prevalence of commuting by walking or bicycling to work for each city. The commuting variables were heavily skewed toward low values, thus were log-transformed in regression analyses. Interactions were assessed between parking variables and population density.

## Results

Most of the cities in the parking database were large metropolitan areas (Table 1): the median population was 303,871 (interquartile range: 467,558) and the median population density was 3,526 people/mile^2^. The median prevalence of walking to work (3.5%) was much higher than that of bicycling (0.8%).

**Table 1:** Descriptive Statistics for Cities in the Drexel University Central Business District Public Parking Survey, 2009

Characteristic	N	Median (Interquartile Range)
**Demographics^a^**		
Population, n	107	303,871 (467,558)
Population density, n/mile^2^	107	3,526.2 (3,861.0)
Median age, y	107	33.7 (3.3)
Male, %	107	48.6 (1.5)
Non-Hispanic white, %	107	60.5 (23.3)
Average household size, n	107	2.38 (0.4)
Median annual family income, $	107	54,721 (16946)
Families at or below poverty level, %	107	14.6 (7.8)
**Parking prices^b^**		
On-street maximum hourly, $	95	1.00 (0.75)
Off-street hourly, $	96	3.00 (3.65)
Off-street daily, $	90	11.38 (7.50)
**Commute mode^c^**		
Walk, %	107	3.5 (3.6)
Bicycle %	107	0.8 (1.4)
Either, %	107	4.8 (4.5)

a 2010 US decennial census data were used for population, age, sex, race/ethnicity, and household size, and 2009 American Community Survey data (9) were used for median annual family income, and poverty level.

b Drexel University Central Business District Parking Survey, 2009 (6).

c 2009 American Community Survey.

There were weak but significant (*P* < .05) correlations between daily off-street and hourly on-street parking prices and the 3 commuting variables (Spearman’s ρ = 0.22–0.31). Off-street hourly prices were not correlated with any of the commuting variables.

Regression analyses focused on daily off-street parking (n = 90 cities) because bivariate correlations were highest for this variable and it is most relevant to full-time workers. In the unadjusted model (Table 2, model 1), daily off-street parking price was associated with walking to work; for every additional dollar charged, the prevalence of walking to work was 3.9% higher (*P* < .001, *R* ^2^ = 0.24). After adjustment for average household size (Table 2, model 2), the association between parking price and walking differed by population density (*P* for interaction = .027). In cities with high population density (≥3,526 people/mile^2^), the prevalence of walking to work was 3.1% higher for every additional dollar charged (*P* < .001); in cities of low population density there was no association (*P* = .98).

**Table 2:** Results of Weighted^a^ Least Squares Linear Regression Between Daily Off-Street Parking Prices and Commute Mode, 90 US Cities^b^

Outcome/Model	Parking Price Variable Statistics		Model Statistics	
	β^c^ (SE)	Wald *P*	Adjusted *R* ^2^	*F* Test *P*
**% Walk to work (log)**				
**Model 1 — unadjusted**	0.039 (0.007)	<.001	0.24	<.001
**Model 2 — adjusted^d^**				
Low population density	−0.0002 (0.012)	.98	0.57	<.001
High population density	0.031 (0.007)	<.001		
**% Bicycle to work (log)**				
**Model 1 — unadjusted**	0.038 (0.014)	.01	0.07	.01
**Model 2 — adjusted^e^**	0.003 (0.014)	.85	0.27	<.001

Abbreviation: SE, standard error.

a Models weighted by 1 ÷ standard error of the average percentage commuting estimate by mode for each city.

b Parking price information from Drexel University Central Business District Public Parking Survey, 2009 (6); prevalence of walking obtained from 2009 American Community Survey (9).

c Dependent variables were log (natural) transformed; 100 × β = percentage change.

d Adjusted for binary population density (either above or below the median population density of 3,526 people/mile^2^) and average household size.

e Adjusted for binary population density (either above or below the median population density of 3,526 people/mile^2^) and median annual family income.

In the unadjusted model, daily off-street parking price was significantly associated with bicycling to work; for every additional dollar charged to park, the prevalence of bicycling to work was 3.8% higher (Table 2, model 1, *P* = .01). After adjustment for median annual family income and population density (Table 2, model 2), there was no association between parking prices and prevalence of bicycling (*P* = .85) and no evidence of an interaction between daily off-street parking price and population density on the association with bicycling to work (*P* = .29).

## Discussion

This is the first multicity ecological study to investigate the association between parking prices and active commuting. After adjustment, walking to work was significantly associated with parking prices among densely populated cities. Although preliminary and ecological in nature, these results hint at the potential for parking policies to influence commuting mode choice. If confirmed, transportation planners could consider this influence when evaluating parking policies, and public health professionals could consider this influence for increasing physical activity.

The interaction between population density and parking prices may be explained by distance between residences and places of employment. More densely populated cities have greater concentrations of residences and employment centers per unit area compared with less densely populated cities (10). Therefore, the distance between residences and places of employment could be shorter, which would allow more walking for transportation (11) and enable parking prices to exert an effect on walking behaviors, but this conjecture cannot be proven in this data set. Bicycling may have been too rare in this sample of cities to detect a similar association.

These preliminary, ecological data should be interpreted cautiously. Individual-level parking price and commute mode data from multiple locations are needed for further study. Adding trip-level parking price assessment to national or local travel surveys is one potential avenue of future research, similar to the assessment of trip-specific tolls in the National Household Travel Survey. Additionally, cities that change parking policies and prices offer the opportunity for natural experiments in this area.

These results suggest a city-level association between parking prices and walking to work in densely populated US cities. City officials, planners, and business owners might consider the potential health behavior-related consequences, both intended and unintended, of changing parking prices. Should future research suggest a causal relation, parking prices could be a policy lever for influencing population physical activity.
